# Whole-Genome Sequencing for Investigating a Health Care-Associated Outbreak of Carbapenem-Resistant *Acinetobacter baumannii*

**DOI:** 10.3390/diagnostics11020201

**Published:** 2021-01-29

**Authors:** Sang Mee Hwang, Hee Won Cho, Tae Yeul Kim, Jeong Su Park, Jongtak Jung, Kyoung-Ho Song, Hyunju Lee, Eu Suk Kim, Hong Bin Kim, Kyoung Un Park

**Affiliations:** 1 Department of Laboratory Medicine, Seoul National University Bundang Hospital, Seongnam 13620, Korea; smilemee@gmail.com (S.M.H.); mdmicrobe@gmail.com (J.S.P.); 2 College of Medicine, Seoul National University, Seoul 03080, Korea; joys0214@snu.ac.kr (H.W.C.); unsymmetry@gmail.com (J.J.); khsongmd@gmail.com (K.-H.S.); yonathan@hanafos.com (H.L.); hbkimmd@snu.ac.kr (E.S.K.); mdopd@hanmail.net (H.B.K.); 3 Department of Laboratory Medicine and Genetics, Samsung Medical Center, Seoul 06351, Korea; voltaire0925@gmail.com; 4 Department of Internal Medicine, Seoul National University Bundang Hospital, Seongnam 13620, Korea; 5 Department of Pediatrics, Seoul National University Bundang Hospital, Seongnam 13620, Korea

**Keywords:** *Acinetobacter baumannii*, whole-genome sequencing, multilocus sequence typing, single nucleotide variant analysis, repetitive sequence polymerase chain reaction

## Abstract

Carbapenem-resistant *Acinetobacter baumannii* (CRAB) outbreaks in hospital settings challenge the treatment of patients and infection control. Understanding the relatedness of clinical isolates is important in distinguishing outbreak isolates from sporadic cases. This study investigated 11 CRAB isolates from a hospital outbreak by whole-genome sequencing (WGS), utilizing various bioinformatics tools for outbreak analysis. The results of multilocus sequence typing (MLST), single nucleotide polymorphism (SNP) analysis, and phylogenetic tree analysis by WGS through web-based tools were compared, and repetitive element polymerase chain reaction (rep-PCR) typing was performed. Through the WGS of 11 *A. baumannii* isolates, three clonal lineages were identified from the outbreak. The coexistence of *bla_OXA-23_, bla_OXA-66_, bla_ADC-25_,* and *armA* with additional aminoglycoside-inactivating enzymes, predicted to confer multidrug resistance, was identified in all isolates. The MLST Oxford scheme identified three types (ST191, ST369, and ST451), and, through whole-genome MLST and whole-genome SNP analyses, different clones were found to exist within the MLST types. wgSNP showed the highest discriminatory power with the lowest similarities among the isolates. Using the various bioinformatics tools for WGS, CRAB outbreak analysis was applicable and identified three discrete clusters differentiating the separate epidemiologic relationships among the isolates.

## 1. Introduction

Carbapenem-resistant *Acinetobacter baumannii* (CRAB) is an important pathogen in healthcare-associated infections and leads to high mortality, especially in intensive care units [1]. *A. baumannii* can cause various conditions, such as bacteremia, bloodstream and surgical wound infections, and ventilator-associated pneumonia [2]. The emergence of multidrug-resistant *A. baumannii* is a challenge in the treatment of patients and infection control since it has the ability to survive on the surface of plastics and can easily spread in the hospital environment [3]. CRAB is designated a critical-priority pathogen by the World Health Organization for the development of new antimicrobial drugs [4]. Currently, only a few therapeutic options, including colistin and tigecycline, are available for CRAB with increasing resistance rates, which is a concerning situation [5].

A well-validated workflow of rapid screening, antimicrobial resistance (AMR) analysis, and accurate strain typing is required for managing antimicrobial resistant strains in healthcare-associated infections [6]. Typing clinical isolates by suitable methods allows health professionals to distinguish outbreak isolates from sporadic or endemic isolates and design rational pathogen control methods such as isolation, cleaning, and/or removal of sources of infection [7].

Historically, strain typing has been based on detecting certain sequence variations in certain genes, i.e., the targeted DNA-based approach [8,9]. This approach includes plasmid DNA analysis, pulsed-field gel electrophoresis (PFGE), conventional multilocus sequence typing (MLST), and repetitive element sequence-based polymerase chain reaction (rep-PCR). These methods investigate small fragments of the bacterial genome, requiring species-dependent protocols, but whole-genome sequencing (WGS) allows resolution in highly related lineages without the need for species-dependent protocols, giving information on pathogen identification, virulence factors, drug susceptibility and comparative genomics [10,11]. Thus, WGS-based analysis has been incorporated in the detection and investigation of outbreaks in recent years [12,13].

While WGS has become cheaper and more reliable due to the development of next-generation sequencing, utilizing WGS data remains challenging in outbreak analysis [14,15] due to the variability in sequencing techniques, bioinformatics tools, different criteria for interpretation, lack of standardization in protocols, and time limits [16,17]. Recently, many web-based tools have been developed for analyzing bacterial pathogens with WGS [10,18,19].

In this study, we aimed to characterize CRAB isolates from a hospital outbreak in a tertiary hospital by WGS, utilizing various bioinformatics tools for outbreak analysis. We provide a comparative analysis of the single nucleotide variant (SNV) approach, where single nucleotide differences compared to a reference genome are identified and compared among isolates, and the MLST approach, where variations in loci of genes are compared among isolates. We aimed to determine the relatedness between the outbreak isolates through SNV- and MLST-based WGS results, further investigate AMR, and compare the results with clinical information and rep-PCR.

## 2. Materials and Methods

This study investigated 11 *A. baumannii* isolates collected in 2015 and 2016 at Seoul National University Bundang Hospital; 9 of the isolates were obtained from the intensive care unit (ICU) and Ward A during a hospital outbreak in 2016, and 2 of the isolates were from patients with CRAB from 2015 in the same institution (Table 1). This study was approved by the institutional review board of Seoul National University Bundang Hospital (B-2008-628-305) on 21 July 2020 with a waiver of informed consent.

In 2016, five patients who stayed in the ICU were diagnosed with CRAB, one of whom was transferred to Ward A. Environmental screenings (bedrails, suction catheters, and central station keyboard) of the ICU and Ward A were included. Acinetobacter isolates were identified by Gram stain morphology, and antibiotic susceptibility (AST) was evaluated with the Vitek2 system (bioMerieux, Marcy l’Etoile, France). The AST results were interpreted based on the Clinical and Laboratory Standards Institute (CLSI) M100 guidelines [20].

### 2.1. Whole-Genome Sequencing

WGS was performed using an Illumina HiSeq4000 (Illumina, San Diego, CA, USA). The results were randomly labeled for this study (#1–#12, excluding #10). Clinical information was not given until the analysis was completed. The whole-genome sequences of eleven *A. baumannii* isolates were assembled and annotated using the Comprehensive Genome Analysis service with the default parameters on the Pathosystems Resource 127 Integration Center (PATRIC) 3.6.6 supported by the National Institutes of Health (https://www.patricbrc.org) [21]. The genome was annotated via Rapid annotation using subsystem technology tool kit (RASTtk) [22] and was assigned a unique genome identifier. Pathogenwatch, developed by the Center for Genomic Pathogen Surveillance (https://pathogen.watch), was additionally utilized for genome assembly [18]. FASTQ files were uploaded and assembled, and the organism was predicted. De novo assembly was additionally performed with Bionumerics 7.6 (Applied Maths, Sint-Martens-Latem, Belgium) using the default settings.

#### 2.1.1. Multilocus Sequence Typing

Conventional MLST was performed on the Pathogenwatch online server. Pathogenwatch uses MLST schemes provided by PubMLST. A sequence type (ST) code is generated based on the combination of detected alleles for *gltA*, *gyrB*, *gdhB*, *recA*, *cpn60*, *gpi*, and *rpoD* with the Oxford scheme [23] and *cpn60*, *fusA*, *gltA*, *pyrG*, *recA*, *rplB*, and *rpoB* with the Pasteur scheme [24].

Core genome (cg) MLST clustering was performed on Pathogenwatch, where profiles are clustered by calculating distances between each assembly that shares a given cgMLST scheme based on 2390 targets for *A. baumannii*. The distance is calculated as the number of different loci for the scheme, ignoring any that are missing. These were then clustered using single linkage clustering based on the calculated pairwise distances in Pathogenwatch based on https://www.cgmlst.org/ncs/schema/3956907/.

Whole-genome MLST (wgMLST) was performed with Bionumerics 7.6 (Applied Maths), where 5619 loci for *A. baumannii* were included for analysis.

#### 2.1.2. Single Nucleotide Variation Analysis

SNV analysis was conducted using the variation analysis service of the PATRIC online server. Isolate #1, which was a non-outbreak isolate from 2015, was selected as the reference genome for the analysis. Burrows-Wheeler Aligner-mem was used for the aligner, and FreeBayes was used for the SNV caller. Only SNVs reported to have a “high” effect, nonsense and frameshift variants, were filtered. Synonymous or missense variants were neglected.

Whole-genome single nucleotide polymorphism (wgSNP) analysis was performed with Bionumerics 7.6 (Applied Maths) using Isolate #1 as the reference genome, and “Strict SNP filtering” was applied.

#### 2.1.3. Cluster Analysis

A phylogenetic tree was created using the Phylogenetic Tree Building Service of the PATRIC online server with the codon tree method. The codon tree method selects single-copy PATRIC PGFams and analyses aligned proteins and coding DNA from single-copy genes using Randomized axelerated maximum likelihood (RAxML) (v8.2.11). One hundred genes were utilized, and the allowed deletions and duplications were set as 0, the default value.

Cluster analysis was performed on the wgMLST and wgSNP data with Bionumerics 7.6 using categorical differences with a scaling factor of 100. Clustering was performed with the unweighted pair group method with arithmetic mean.

#### 2.1.4. Antimicrobial Resistance Prediction

AMR was predicted by ResFinder 4.0 [25] with the default settings, threshold for %ID as 90% and minimum length of 60%, and KmerResistance 2.2, using the species determination on maximum query coverage, select identity threshold as 70%, and threshold for depth corr as 10% [26,27].

### 2.2. Repetitive Element PCR

DNA was extracted with an UltraClean microbial DNA isolation kit (MoBio Laboratories, Carlsbad, CA, USA), and the concentration was assessed with a NanoDrop. DNA was amplified with a DiversiLab Acinetobacter kit (bioMerieux, France) following the manufacturer’s instructions. Analysis was performed with web-based DiversiLab software using the Pearson correlation (PC) method, which weighs peak intensities; the Kullback–Leiber (KL) method, which weighs the absence of peaks; and the extended Jaccard (XJ) method, which is sensitive to the presence of peaks, for analysis. In this study, strains with greater than 95 % similarity were considered similar, and strains with less than 95% similarity were considered different. We analyzed the strain diversity of 11 isolates in two batches, with two isolates commonly included in both batches and eight other non-outbreak isolates included in the rep-PCR analysis.

## 3. Results

This study comprised of 11 *A. baumannii* isolates collected in 2015 and 2016; nine isolates were from a hospital outbreak in 2016 in the ICU, and two isolates (#1 and #7) were from patients with CRAB from 2015 in the same institution (Table 1). Specimens were from blood (*n* = 3), sputum (*n* = 2), transtracheal aspirate (*n* = 2), and surveillance culture/swab (*n* = 4). The AMR results from the specimens (*n* = 11) showed carbapenem resistance.

### 3.1. Whole-Genome Sequencing Results

From WGS, the isolates were assigned as *A. baumannii*. Due to the difference in assembly, the genome lengths and contigs varied according to different bioinformatics tools (Table S1).

#### 3.1.1. MLST

**Figure 1:**
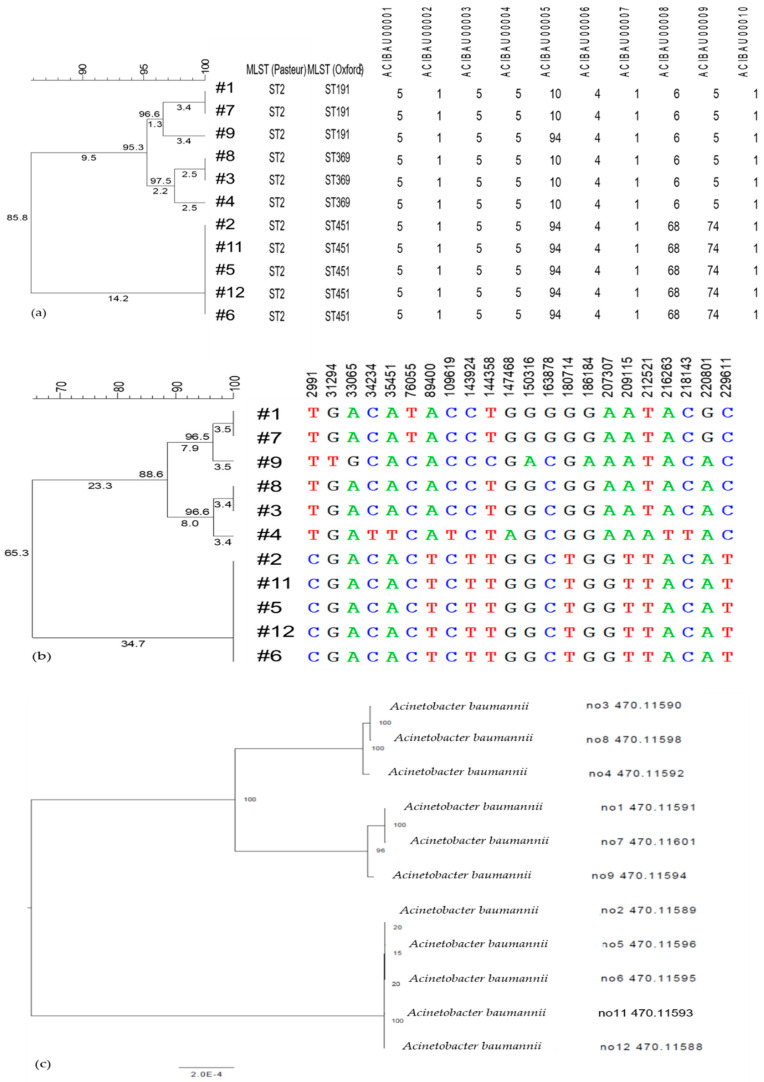
wgMLST and wgSNP, SNV dendrogram of 11 isolates: (**a**) wgMLST; (**b**) wgSNP by Bionumerics; and (**c**) SNV analysis by PATRIC. Not all the loci are shown in (**a**,**b**).

The isolates were not distinguished by the MLST Pasteur scheme, where all isolates were typed as ST 2. However, through the MLST Oxford scheme, Isolates #2, #5, #6, #11, and #12 were assigned as ST451; #3, #4, and #8 as ST369; and #1, #7, and #9 as ST191 (Figure 1a).

**Figure 2:**
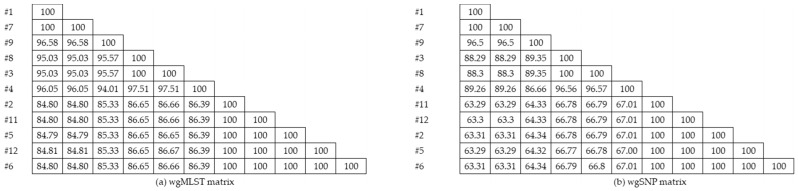
(**a**) wgMLST and (**b**) wgSNP similarity matrix of isolates.

Based on the cgMLST scheme with 2390 targets on Pathogenwatch, at the threshold of 10, isolates were clustered into three groups of #2, #6, #11, #12, and #5; #1 and #7; and #3 and #8. Isolates #4 and #9 were not clustered with any other isolates. At the threshold of 30, #4 was clustered with #3 and #8, but #9 remained separate from the other isolates. The de novo assembled genomes submitted to the calculation engine by BioNumerics produced a wgMLST profile with 5619 loci. The comparison of characteristics showed similarity values of 84.81–100% for the isolates (Figure 2).

#### 3.1.2. Single Nucleotide (SNV/SNP) Analysis

Through PATRIC SNV analysis, 10,474 variants were identified, and 47 high-effect SNVs were present, comprising 22 frameshift variants and 25 nonsense variants, compared to reference Isolate #1. The number of SNVs ranged from 0 to 25 (median = 22.5). Isolate #7, which was another non-outbreak isolate from 2015, did not show any high-effect SNVs. Isolate sets with less than three SNV differences and sharing more than 90% of SNVs were grouped as follows: #1 and #7; #2, #5, #6, #11, and #12; and #3 and #8. Isolate #4 showed similar SNV profiles with #3 and #8, sharing SNVs in 88.9% and 83.3%, respectively, and Isolate #9 showed 12 SNVs, but the shared SNVs were less than 66.7% with the other isolates.

The wgSNP analysis identified 0–3671 SNPs compared to the reference Isolate #1, with a median of 2420 SNPs (Figure 1b). Similarities ranged from 63.29% to 100% for the 10 isolates (Figure 2), in which, compared to the reference, Isolate #7 did not show any SNP differences with strict filtering. Isolates #2, #5, #6, #11, and #12 showed 1–2 SNP differences, and Isolates #3 and #8 showed only one SNP difference.

#### 3.1.3. Cluster Analysis

Through cluster analysis, two main clusters with three clones were identified, where Isolates #1, #7, #9, #8, #3, and #4 were in one cluster and Isolates #2, #5, #6, #11, and #12 were in the other. Among the first cluster, Isolates #1, #7, and #9 were grouped, and the other included Isolates #8, #3, and #4. Isolates #1 and #7; #3 and #8; and #2, #11, #5, #12, and #6 showed similarity indices of 100%. As with wgSNP, the clustering dendrogram showed two main clusters with three clones, as in wgMLST.

The phylogenetic tree created with PATRIC also showed three clones with two main clusters: #3, #4, and #8; #1, #7, and #9; and #2, #5, #6, #11, and #12 (Figure 1c). The sequences of Isolates #1 and #7 were identical, those of #3 and #8 were identical, and those of #12, #5, #2, #6, and #11 were identical to each other. The dendrograms created with PATRIC and Bionumerics all showed three clones with differences in the similarity index.

#### 3.1.4. Antimicrobial Resistance Prediction

The antibiotic susceptibility testing results were predicted through in silico analysis of resistance determination by ResFinder and KmerResistance (Table 2). All of the isolates showed coexistence of the OXA carbapenemase genes *bla_OXA-23_* and *bla_OXA-66_* and the Acinetobacter-derived cephalosporinase gene *bla_ADC-25_*, while six isolates had the additional gene *bla_TEM-1D_*, and these results were concordant with ResFinder and KmerResistance.

Six different aminoglycoside-inactivating enzymes and their variants were detected. Aminoglycoside acetyltransferase (AAC) *aac(6′)-Ib-cr* was found in six isolates assigned as MLST (Oxford) ST191 and ST369. Three variants of aminoglycoside phosphotransferase, *aph(3′)Ia*, *aph(3″)Ib*, and *aph(6)-Id*, were identified in the other five isolates, and all 11 isolates had variants in *armA*, a 16S rRNA methylase gene. The *aac(6′)-Ib-cr* variant was identified only with ResFinder.

The sulfonamide resistance gene sul1 was present in six isolates, and *sul2* was present in the other remaining isolates. The phenicol resistance gene *catB8* was present in six isolates with *sul1*. ResFinder and Kmer resistance showed different results for the macrolide resistance gene *mphE*, where all isolates were predicted to have this gene in KmerResistance, whereas ResFinder showed that only nine isolates had this. All of the isolates were predicted to be susceptible to colistin.

Two distinct patterns of antimicrobial resistance genes were present among the isolates and it showed correlation with the other clustering methods, where there were two main clusters by wgMLST, wgSNP, and SNV analysis. Isolates #2, #5, #6, #11, and #12, showed beta-lactam resistance genes of *bla*_OXA-23_, *bla*_OXA-66_, *bla*_ADC-25_, and *bla*_TEM-1D_; aminoglycoside resistance genes *aph(3′)-Ia*, *aph(6)-Id*, and *aph(3″)-Ib*, *armA*; sulfonamide gene *sul2*; macrolide resistance genes *msr(E)* and *mph(E)*; and tetracycline resistance gene *tet(B),* and theses were grouped together as a cluster. For Isolates #1, #7, #9, #8, #3, and #4, two clusters were formed by wgMLST, wgSNP, and SNV analysis, and all of the resistance genes were concordant for theses isolates, except for *mph(E)*, which was detected additionally by KmerResistance for Isolates #3 and #8, which were clustered together as identical by wgMLST, wgSNP, SNV, and repPCR.

### 3.2. Rep-PCR

**Figure 3:**
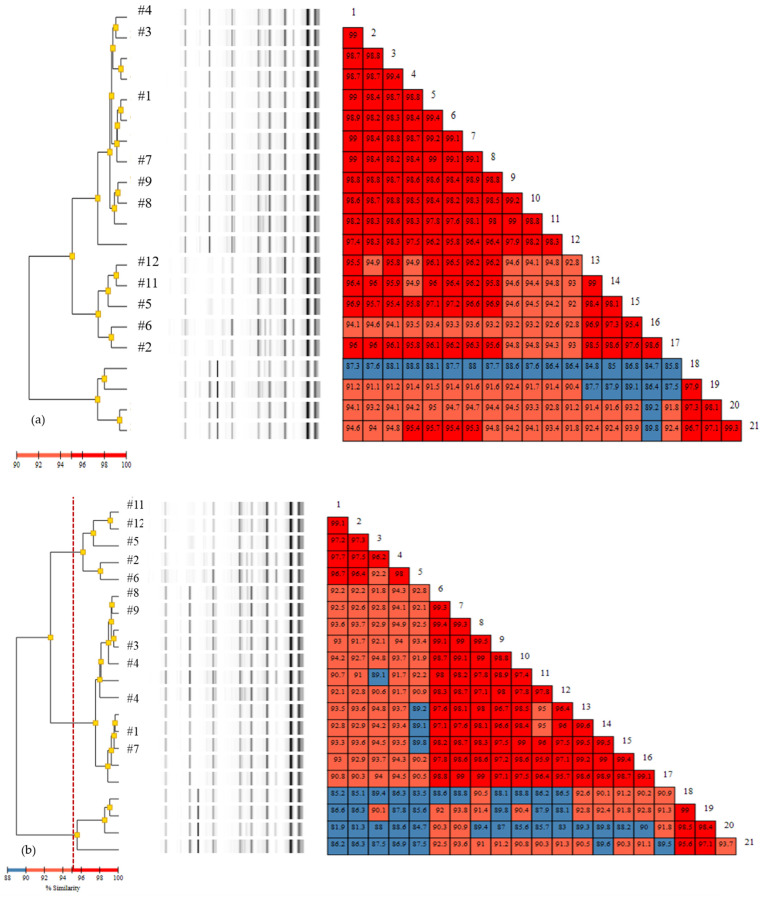

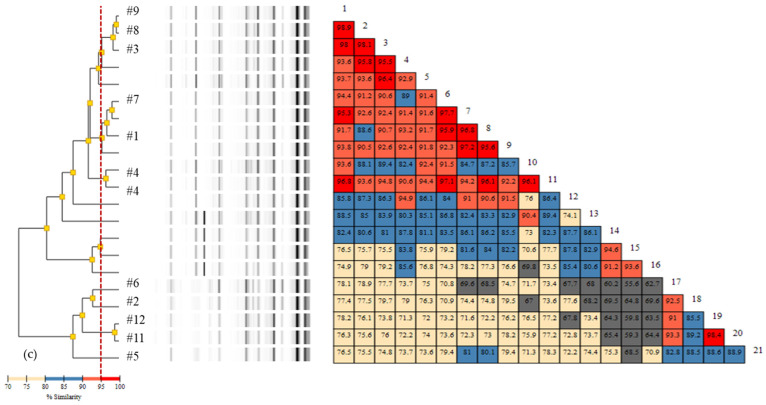
Repetitive element-PCR results by: (**a**) Pearson correlation; (**b**) Kullback–Leiber; and (**c**) extended Jaccard method.

Rep-PCR typing results varied by the analysis methods. The 11 isolates were clustered into four clones (#1 and #7; #3 and #4; #8 and #9; and #12, #11, #5, #6, and #2), by the PC method and three clones (#2, #5, #6, #11, and #12; #3, #4, #8, and #9; and #1 and #7), by the KL method (Figure 3). However, with the XJ method, the similarities were lower, creating three clusters (#3, #8, and #9; #1 and #7; and #2, #5, #6, #11, and #12), with Isolate #4 not being clustered with the other isolates. Clusters including Isolates #2, #5, #6, #11, and #12 were commonly present with the PC and KL methods, showing >95% similarities among the isolates.

### 3.3. Comparison of Typing Methods

The phylogenetic tree created with WGS using various tools showed similar clustering (#2, #5, #6, #11, and #12) to that with rep-PCR (Figure 2; Figure 3). For Isolates #1, #3, #4, #7, #8, and #9, the clusters varied among the methods, and, with the WGS phylogenetic tree, these isolates were separated once more into two clusters (#3, #4, and #8 and #1, #7, and #9). wgSNP and wgMLST also identified isolates in the same clusters; however, the similarity values were lower for wgSNP among the isolates.

## 4. Discussion

Outbreak analysis of 11 *A. baumannii* isolates was performed with WGS and rep-PCR, and three clonal lineages were identified from the outbreak. The coexistence of *bla_OXA-23_*, *bla_OXA-66_*, *bla_ADC-25_*, and *armA* with additional aminoglycoside-inactivating enzymes, predicted to confer multidrug resistance, was identified in all the isolates.

The application of WGS allowed clustering analysis of *A. baumannii* in an outbreak and wgMLST, SNV/wgSNP, and phylogenetic tree analyses. WGS showed three clusters, Cluster 1 (#2, #5, #6, #11, and #12) with outbreak isolates from 2016, Cluster 2 (#3, #4, and #8) with outbreak isolates from 2016, and Cluster 3 (#1, #7, and #9) with two non-outbreak isolates from 2015.

Although both the MLST Pasteur and Oxford schemes were based on seven loci, the Pasteur scheme could not differentiate the isolates, but the Oxford scheme was capable of differentiating the closely related isolates due to the greater number of sequence types [28,29]. However, *A. baumannii* has both high gene content variation [30] and substantial levels of recombination [31], suggesting insufficient discrimination among isolates, requiring a higher resolution for WGS [28,29]. ST191, ST369, and ST451 were identified in the isolates; these ST types are frequently isolated in Korea and other Asian countries [32,33] and thus could not discriminate the clones within the outbreak.

Clustering and phylogenetic analysis from wgMLST and wgSNP showed three clusters, and SNV analysis compared the similarities among the strains with high-effect SNVs. The discriminatory power was highest with the SNV analysis, but since the number of isolates was small, clustering results did not vary among the different methods. Cluster 1, including Isolates #2, #5, #6, #11, and #12, showed 100% similarity within the isolates by clustering analysis of wgMLST and wgSNP/SNV, suggesting a single origin. Isolate #2 was extracted from transtracheal aspirate specimens of an ICU patient. Isolate #5 was obtained from the keyboard of the main station at the ICU, and Isolate #6 was obtained from the bedrail of Ward A, where the patient from the ICU was transferred to. Isolates #11 and #12 were each obtained from suction catheters at the bedside from the ICU and Ward A, respectively. These results support the spread of the same clone in the ICU and Ward A by the transfer of a patient. In Cluster 2, #3 and #8 showed 100% similarity, suggesting the same clonal origin. Two patients were in the ICU during the same time period. The patients from Cluster 1 also stayed in the ICU during the same period as the patients in Cluster 2, suggesting multiple clones during the outbreak. Although #4 was clustered with #3 and #8, it showed similarities of 97.5% by wgMLST and 96.6% by wgSNP, and SNV analysis with PATRIC showed a similarity of 72.7% to #3, suggesting a different clone. A previous study suggested a threshold of 2.5 SNPs to distinguish outbreak isolates from non-outbreak isolates in *A. baumannii* [34], and the isolate sets with less than three SNV differences and sharing more than 90% of SNVs were #1 and #7; #2, #5, #6, #11, and #12; and #3 and #8, where the 2016 outbreak isolates were mainly grouped into two clones.

Despite the complexity of WGS, we were able to analyze WGS, including MLST, SNV and phylogenetic tree analysis, all commonly utilized for outbreak analysis, with the web-based public tools Pathogenwatch and PATRIC [11,21,35]. We used the DiversiLab system for rep-PCR and showed similar results to the WGS phylogenetic tree and core genome clustering, as previously reported [36,37]. rep-PCR can be performed in a relatively short time without extensive post-experiment analyses, but the exact sequences are not available, and variabilities are reported for interlaboratory comparison of fingerprints [38,39]. WGS has the advantage of reanalyzing the results with different sequence types and can provide additional information on AMR, virulence genes and transmission scenarios [10,40]. WGS has sufficient resolution to determine transmission within clonal outbreaks [34,37]. In most cases of outbreak investigation, the source case is not identified, and genomic variability increases over time, making it hard to determine the threshold point [41]. However, it should be noted that the “significance” of the difference between isolates should be judged based on a comprehensive understanding of the genetics and epidemiology of the pathogen, the setting within which the issue is being studied, and the tools being used in the investigation [41].

AMR prediction with ResFinder and KmerResistance showed high concordance, with most of the resistance genes identified commonly. The isolates showed a common antibiotic resistance profile reported in multidrug-resistant *A. baumannii* in Korea [32,42,43]. A previous study showed high concordance with ResFinder, an assembly-based tool, and KmerResistance, a read-based tool, in high-quality sequencing results, as in our study [26]. False-positive predictions through sequencing-based methods may be possible, requiring caution for interpretation [18,44]. Excluding the discrepancy of one isolate for resistance of sulfonamide gene and the discrepancies for prediction of quinolone resistance for five isolates, CRABs from the outbreak showed resistance to all the above antimicrobial agents except for colistin and the results correlated with the in silico prediction results. Only few comprehensive studies have investigated the concordance between the prediction for resistance by WGS and the conventional phenotypic antimicrobial susceptibility testing [45]. Previous studies have shown various concordance rates varying by antimicrobial agents [46,47]. The discordance of prediction with fluroquinolone resistance has been reported regarding the *aac(6′)-Ib-cr*, where an non-wild type is linked to specific sequences only [25] and the fluoroquinolone resistance are predicted through other genes including *gyrA, parC,* not included in the prediction tool, ResFinder [25,47]. The high concordance of genotype-phenotype correlation for antimicrobial resistance excluding the fluoroquinolone in our study, is possibly due to the inclusion of isolates within a single outbreak, which showed multi-resistance to the antimicrobial agents, where multi-resistance of *A. baumannii* is common in Korea. If various CRABs from different clinical background had been included, then the prediction may have showed discrepancies among the antimicrobial agents.

Despite the wide applicability of the WGS in outbreak analysis, the cost of WGS is still expensive, considering the equipment set up, cost of sequencing and bioinformatics analysis [48]. There are few studies evaluating the cost-effectiveness of bacterial WGS surveillance compared to the standard of care in detecting hospital outbreaks. Kumar et al. suggests that preliminary studies show WGS surveillance could be a cost-effective strategy [49,50]. However, clinical settings vary and cost-effectiveness has only been studied in certain organisms such as *Klebsiella pneumoniae* and *Pseudomonas aeruginosa*, thus more economical assessment would be necessary.

Although our study was a retrospective study, when WGS is performed during an outbreak, it may inform appropriate patient isolation protocols that could aid in the control of an outbreak [10]. Through recent advances in bioinformatics tools for WGS, outbreak analysis can be performed in a relatively short time. We characterized the clonal clusters in a nosocomial outbreak of CRAB in a tertiary hospital with WGS, supporting the use of WGS in healthcare infection epidemiologic studies.

## Figures and Tables

**Table 1:** Clinical information of *A. baumannii* isolates included in this study.

Isolate	Specimen	Date of Isolation	Hospital Stay
#1	TTA	2015-01-20	*
#2	TTA	2016-03-21	ICU
#3	Sputum	2016-03-16	ICU
#4	Blood	2016-04-13	ICU
#5	Keyboard	2016-03-25	ICU
#6	Bed rail	2016-03-28	Ward A
#7	Blood	2015-01-20	*
#8	Blood	2016-03-15	ICU
#9	Sputum	2016-03-10	ICU
#11	Suction catheter	2016-03-25	ICU
#12	Suction catheter	2016-03-28	Ward A

Abbreviations: TTA, transtracheal aspirate; ICU, intensive care unit. * Non-outbreak isolates from 2015.

**Table 2:** In silico detection of resistance determinants of antimicrobial agents and the antibiogram from VitekII.

Isolate	ST	Beta-Lactam Resistance Genes	TZP	CAZ	FEP	IPM	MER	Aminoglycoside Resistance Genes	AMK	GEN	TOB	Sulfonamide Genes	SXT	Quinolone Genes	CIP	Phenicol Genes	Macrolide	Tetracycline Genes	CST
#1	191	*bla*_OXA-23_, *bla*_OXA-66_, *bla*_ADC-25_	*R*	*R*	*R*	*R*	*R*	aadA1, aac(6′)-Ib-cr *, armA	*R*	*R*	*R*	*sul1*	*R*	*aac(6′)-Ib-cr*	*R*	*catB8*	*msr(E). mph(E)*		*S*
#2	451	*bla*_OXA-23_, *bla*_OXA-66_, *bla*_ADC-25_, *bla*_TEM-1D_	*R*	*R*	*R*	*R*	*R*	*aph(3′)-Ia, aph(6)-Id, aph(3* *″)-Ib, armA*	*R*	*R*	*R*	*sul2*	*R*		*R*		*msr(E). mph(E)*	*tet(B)*	*S*
#3	369	*bla*_OXA-23_, *bla*_OXA-66_, *bla*_ADC-25_	*R*	*R*	*R*	*R*	*R*	aadA1, aac(6′)-Ib-cr *, armA	*R*	*R*	*R*	*sul1*	*R*	*aac(6′)-Ib-cr*	*R*	*catB8*	msr(E), mph(E) **		*S*
#4	369	*bla*_OXA-23_, *bla*_OXA-66_, *bla*_ADC-25_	*R*	*R*	*R*	*R*	*R*	aadA1, aac(6′)-Ib-cr *, armA	*R*	*R*	*R*	*sul1*	*R*	*aac(6′)-Ib-cr*	*R*	*catB8*	*msr(E). mph(E)*		*S*
#5	451	*bla*_OXA-23_, *bla*_OXA-66_, *bla*_ADC-25_, *bla*_TEM-1D_	*R*	*R*	*R*	*R*	*R*	*aph(3′)-Ia, aph(6)-Id, aph(3* *″)-Ib, armA*	*R*	*R*	*R*	*sul2*	*R*		*R*		*msr(E). mph(E)*	*tet(B)*	*S*
#6	451	*bla*_OXA-23_, *bla*_OXA-66_, *bla*_ADC-25_, *bla*_TEM-1D_	*R*	*R*	*R*	*R*	*R*	*aph(3′)-Ia, aph(6)-Id, aph(3* *″)-Ib, armA*	*R*	*R*	*R*	*sul2*	*R*		*R*		*msr(E). mph(E)*	*tet(B)*	*S*
#7	191	*bla*_OXA-23_, *bla*_OXA-66_, *bla*_ADC-25_	*R*	*R*	*R*	*R*	*R*	aadA1, aac(6′)-Ib-cr *, armA	*R*	*R*	*R*	*sul1*	*S*	*aac(6′)-Ib-cr*	*R*	*catB8*	*msr(E). mph(E)*		*S*
#8	369	*bla*_OXA-23_, *bla*_OXA-66_, *bla*_ADC-25_	*R*	*R*	*R*	*R*	*R*	aadA1, aac(6′)-Ib-cr *, armA	*R*	*R*	*R*	*sul1*	*R*	*aac(6′)-Ib-cr*	*R*	*catB8*	msr(E), mph(E) **		*S*
#9	191	*bla*_OXA-23_, *bla*_OXA-66_, *bla*_ADC-25_	*R*	*R*	*R*	*R*	*R*	aadA1, aac(6′)-Ib-cr *, armA, ant(3”)-Ia	*R*	*R*	*R*	*sul1*	*R*	*aac(6′)-Ib-cr*	*R*	*catB8*	*msr(E). mph(E)*		*S*
#11	451	*bla*_OXA-23_, *bla*_OXA-66_, *bla*_ADC-25_, *bla*_TEM-1D_	*R*	*R*	*R*	*R*	*R*	*aph(3′)-Ia, aph(6)-Id, aph(3* *″)-Ib, armA*	*R*	*R*	*R*	*sul2*	*R*		*R*		*msr(E). mph(E)*	*tet(B)*	*S*
#12	451	*bla*_OXA-23_, *bla*_OXA-66_, *bla*_ADC-25_, *bla*_TEM-1D_	*R*	*R*	*R*	*R*	*R*	*aph(3′)-Ia, aph(6)-Id, aph(3* *″)-Ib, armA*	*R*	*R*	*R*	*sul2*	*R*		*R*		*msr(E). mph(E)*	*tet(B)*	*S*

Abbreviations: ST, strain type; TZP, piperacillin–tazobactam; CAZ, ceftazidime; FEP, cefepime; IPM, imipenem; MER, meropenem; AMK, amikacin; GEN, gentamicin; TOB, tobramycin; SXT, trimethoprim–sulfamethoxazole; CIP, ciprofloxacin; CST, colistin. * Identified only with ResFinder, ** Identified only with KmerResistance.

## Data Availability

The data presented in this study are available on request from the corresponding author.

## Supplementary Materials

The following are available online at https://www.mdpi.com/2075-4418/11/2/201/s1, Table S1: Whole genome assembly results of 11 *Acinetobacter baumannii* isolates.

## References

[1] Dijkshoorn L., Nemec A., Seifert H. (2007). An increasing threat in hospitals: Multidrug-resistant Acinetobacter baumannii. Nat. Rev. Microbiol..

[2] Weiner L.M., Webb A.K., Limbago B., Dudeck M.A., Patel J., Kallen A.J., Edwards J.R., Sievert D.M. (2016). Antimicrobial-resistant pathogens associated with healthcare-associated infections: Summary of data reported to the National Healthcare Safety Network at the Centers for Disease Control and Prevention, 2011–2014. Infect. Control. Hosp. Epidemiol..

[3] Wendt C., Dietze B., Dietz E., Rüden H. (1997). Survival of *Acinetobacter baumannii* on dry surfaces. J. Clin. Microbiol..

[4] Tacconelli E., Magrini N., Kahlmeter G., Singh N.J. (2017). Global Priority List of Antibiotic-Resistant Bacteria to Guide Research, Discovery, and Development of New Antibiotics.

[5] Isler B., Doi Y., Bonomo R.A., Paterson D.L. (2018). New treatment options against carbapenem-resistant Acinetobacter baumannii infections. Antimicrob. Agents Chemother..

[6] Kalenić S., Budimir A.J. (2009). The role of the microbiology laboratory in healthcare-associated infection prevention. Int. J. Infect. Control..

[7] Tenover F.C., Arbeit R.D., Goering R.V. (1997). How to select and interpret molecular strain typing methods for epidemiological studies of bacterial infections a review for healthcare epidemiologists. Infect. Control. Hosp. Epidemiol..

[8] MacCannell D. (2013). Bacterial strain typing. Clin. Lab. Med..

[9] Li W., Raoult D., Fournier P.E. (2009). Bacterial strain typing in the genomic era. FEMS Microbiol. Rev..

[10] Quainoo S., Coolen J.P., van Hijum S.A., Huynen M.A., Melchers W.J., van Schaik W., Wertheim H.F. (2017). Whole-genome sequencing of bacterial pathogens: The future of nosocomial outbreak analysis. Clin. Microbiol. Rev..

[11] Gilchrist C.A., Turner S.D., Riley M.F., Petri W.A., Hewlett E.L. (2015). Whole-genome sequencing in outbreak analysis. Clin. Microbiol. Rev..

[12] Harris S.R., Cartwright E.J., Török M.E., Holden M.T., Brown N.M., Ogilvy-Stuart A.L., Ellington M.J., Quail M.A., Bentley S.D., Parkhill J.J. (2013). Whole-genome sequencing for analysis of an outbreak of meticillin-resistant Staphylococcus aureus: A descriptive study. Lancet Infect. Dis..

[13] Durand G., Javerliat F., Bes M., Veyrieras J.-B., Guigon G., Mugnier N., Schicklin S., Kaneko G., Santiago-Allexant E. (2018). Bouchiat Routine whole-genome sequencing for outbreak investigations of Staphylococcus aureus in a national reference center. Front. Microbiol..

[14] Köser C.U., Ellington M.J., Cartwright E.J., Gillespie S.H., Brown N.M., Farrington M., Holden M.T., Dougan G., Bentley S.D., Parkhill J.J. (2012). Routine use of microbial whole genome sequencing in diagnostic and public health microbiology. PLoS Pathog..

[15] Rossen J.W., Friedrich A., Moran-Gilad J.J. (2018). Practical issues in implementing whole-genome-sequencing in routine diagnostic microbiology. Clin. Microbiol. Infect..

[16] Bogaerts B., Winand R., Fu Q., Van Braekel J., Ceyssens P.-J., Mattheus W., Bertrand S., De Keersmaecker S.C., Roosens N.H., Vanneste K.J. (2019). Validation of a bioinformatics workflow for routine analysis of whole-genome sequencing data and related challenges for pathogen typing in a European National Reference Center: Neisseria meningitidis as a proof-of-concept. Front. Microbiol..

[17] Fricke W.F., Rasko D.A. (2014). Bacterial genome sequencing in the clinic: Bioinformatic challenges and solutions. Nat. Rev. Genet..

[18] Neher R.A., Bedford T.J. (2018). Real-time analysis and visualization of pathogen sequence data. J. Clin. Microbiol..

[19] Snyder E., Kampanya N., Lu J., Nordberg E.K., Karur H., Shukla M., Soneja J., Tian Y., Xue T., Yoo H.J. (2007). PATRIC: The VBI pathosystems resource integration center. Nucleic Acids Res..

[20] CLSI (2020). Performance Standards for Antimicrobial Susceptibility Testing.

[21] Wattam A.R., Abraham D., Dalay O., Disz T.L., Driscoll T., Gabbard J.L., Gillespie J.J., Gough R., Hix D., Kenyon R.J. (2014). PATRIC, the bacterial bioinformatics database and analysis resource. Nucleic Acids Res..

[22] Brettin T., Davis J.J., Disz T., Edwards R.A., Gerdes S., Olsen G.J., Olson R., Overbeek R., Parrello B., Pusch G.D. (2015). RASTtk: A modular and extensible implementation of the RAST algorithm for building custom annotation pipelines and annotating batches of genomes. Sci. Rep..

[23] Bartual S.G., Seifert H., Hippler C., Luzon M.A., Wisplinghoff H., Rodríguez-Valera F.J. (2005). Development of a multilocus sequence typing scheme for characterization of clinical isolates of Acinetobacter baumannii. J. Clin. Microbiol..

[24] Diancourt L., Passet V., Nemec A., Dijkshoorn L., Brisse S. (2010). The population structure of Acinetobacter baumannii: Expanding multiresistant clones from an ancestral susceptible genetic pool. PLoS ONE.

[25] Bortolaia V., Kaas R.S., Ruppe E., Roberts M.C., Schwarz S., Cattoir V., Philippon A., Allesoe R.L., Rebelo A.-R., Florensa A.F. (2020). ResFinder 4.0 for predictions of phenotypes from genotypes. J. Antimicrob. Chemother..

[26] Clausen P.T., Zankari E., Aarestrup F.M., Lund O. (2016). Benchmarking of methods for identification of antimicrobial resistance genes in bacterial whole genome data. J. Antimicrob. Chemother..

[27] Clausen P.T., Aarestrup F.M., Lund O. (2018). Rapid and precise alignment of raw reads against redundant databases with KMA. BMC Bioinform..

[28] Castillo-Ramírez S., Graña-Miraglia L.J. (2019). Inaccurate multilocus sequence typing of Acinetobacter baumannii. Emerg. Infect. Dis..

[29] Hua X., Zhang L., He J., Leptihn S., Yu Y.J. (2020). Population Biology and Epidemiological Studies of Acinetobacter baumannii in the Era of Whole Genome Sequencing: Is the Oxford Scheme Still Appropriate?. Front. Microbiol..

[30] Graña-Miraglia L., Lozano L.F., Velázquez C., Volkow-Fernández P., Pérez-Oseguera Á., Cevallos M.A., Castillo-Ramírez S.J. (2017). Rapid gene turnover as a significant source of genetic variation in a recently seeded population of a healthcare-associated pathogen. Front. Microbiol..

[31] Snitkin E.S., Zelazny A.M., Montero C.I., Stock F., Mijares L., Murray P.R., Segre J.A. (2011). Genome-wide recombination drives diversification of epidemic strains of Acinetobacter baumannii. Proc. Natl. Acad. Sci. USA.

[32] Lee S.Y., Oh M.H., Yun S.H., Choi C.W., Park E.C., Song H.S., Lee H., Yi Y.S., Shin J., Chung C.J. (2018). Genomic characterization of extensively drug-resistant Acinetobacter baumannii strain; KAB03 belonging to ST451 from Korea. Infect. Genet. Evol..

[33] Yoon E.J., Kim D., Lee H., Lee H.S., Shin J.H., Uh Y., Shin K.S., Kim Y.A., Park Y.S., Shin J.H. (2019). Counter clinical prognoses of patients with bloodstream infections between causative Acinetobacter baumannii clones ST191 and ST451 belonging to the international clonal lineage II. Front. Public. Health.

[34] Fitzpatrick M.A., Ozer E.A., Hauser A.R. (2016). Utility of whole-genome sequencing in characterizing Acinetobacter epidemiology and analyzing hospital outbreaks. J. Clin. Microbiol..

[35] Wattam A.R., Davis J.J., Assaf R., Boisvert S., Brettin T., Bun C., Conrad N., Dietrich E.M., Disz T., Gabbard J.L. (2017). Improvements to PATRIC; the all-bacterial bioinformatics database and analysis resource center. Nucleic Acids Res..

[36] Higgins P.G., Dammhayn C., Hackel M., Seifert H.J. (2010). Global spread of carbapenem-resistant Acinetobacter baumannii. J. Antimicrob. Chemother..

[37] Venditti C., Vulcano A., D’Arezzo S., Gruber C., Selleri M., Antonini M., Lanini S., Marani A., Puro V., Nisii C.J. (2019). Epidemiological investigation of an Acinetobacter baumannii outbreak using core genome multilocus sequence typing. J. Glob. Antimicrob. Resist..

[38] Higgins P.G., Hujer A.M., Hujer K.M., Bonomo R.A., Seifert H.J. (2012). Interlaboratory reproducibility of DiversiLab rep-PCR typing and clustering of Acinetobacter baumannii isolates. J. Med. Microbiol..

[39] Rafei R., Kempf M., Eveillard M., Dabboussi F., Hamze M., Joly-Guillou M.-L. (2014). Current molecular methods in epidemiological typing of Acinetobacter baumannii. Future Microbiol..

[40] Lewis T., Loman N., Bingle L., Jumaa P., Weinstock G., Mortiboy D., Pallen M.J. (2010). High-throughput whole-genome sequencing to dissect the epidemiology of Acinetobacter baumannii isolates from a hospital outbreak. J. Hosp. Infect..

[41] Goering R.V., Köck R., Grundmann H., Werner G., Friedrich A.W., Markers E.S. (2013). From theory to practice: Molecular strain typing for the clinical and public health setting. Euro. Surveill..

[42] Kim M.H., Jeong H., Sim Y.M., Lee S., Yong D., Ryu C.-M., Choi J.Y. (2020). Using comparative genomics to understand molecular features of carbapenem-resistant Acinetobacter baumannii from South Korea causing invasive infections and their clinical implications. PLoS ONE.

[43] Jeon H., Kim S., Kim M.H., Kim S.Y., Nam D., Park S.C., Park S.-H., Bae H., Lee H.-J., Cho J.H. (2018). Molecular epidemiology of carbapenem-resistant Acinetobacter baumannii isolates from a Korean hospital that carry blaOXA-23. Infect. Genet. Evol..

[44] Boolchandani M., D’Souza A.W., Dantas G. (2019). Sequencing-based methods and resources to study antimicrobial resistance. Nat. Rev. Genet..

[45] Ellington M.J., Ekelund O., Aarestrup F.M., Canton R., Doumith M., Giske C., Grundman H., Hasman H., Holden M.T.G., Hopkins K.L. (2017). The role of whole genome sequencing in antimicrobial susceptibility testing of bacteria: Report from the EUCAST Subcommittee. Clin. Microbiol. Infect..

[46] Kumburu H.H., Sonda T.L., van Zwetselaar M., Leekitcharoenphon P., Lukjancenko O., Mmbaga B.T., Alifrangis M., Lund O., Aaresrup F., Kibiki G. (2019). Using WGS to identify antibiotic resistance genes and predict antimicrobial resistance phenotypes in MDR Acinetobacter baumannii in Tazania. J. Antimicrob. Chemother..

[47] Liu F., Zhu Y., Yi Y., Lu N., Zhu B., Hu Y. (2014). Comparative genomic analysis of *Acinetobacter baumannii* clinical isolates reveals extensive genomic variation and diverse antibiotic resistance determinants. BMC Genom..

[48] Kwong J.C., McCallum N., Sintchenko V.L., Howden B.P. (2015). Whole genome sequencing in clinical and public health microbiology. Pathology.

[49] Kumar P., Tech B., Sundermann A.J., Martin E., Snyder G.M., Marsh J.W., Harrison L.H., Roberts M.S. (2020). Method for economic evaluation of bacterial whole genome sequencing surveillance compared to standard of care in detecting hospital outbreaks. Clin. Infect. Dis..

[50] Dymond A., Davies H., Mealing S., Pollit V., Coll F., Brown N.M., Peacock S.J. (2020). Genomic surveillance of methicillin-resistant Staphylococcus aureus: A mathematical early modelling study of cost effectiveness. Clin. Infect. Dis..

