# Supplementary material for: Whole-Genome Sequencing for Investigating a Health Care-Associated Outbreak of Carbapenem-Resistant Acinetobacter baumannii
Source: Diagnostics (Basel). 2021 Jan 29;11(2):201. doi: 10.3390/diagnostics11020201 (PMC7910894; doi:10.3390/diagnostics11020201)
Supplement: Supplementary file 1 [file diagnostics-11-00201-s001.pdf]

Table S1. Whole genome assembly results of 11 *Acinetobacter baumannii* isolates.

**Isolates**	**#1**	**#2**	**#3**	**#4**	**#5**	**#6**	**#7**	**#8**	**#9**	**#11**	**#12**
PATRIC											
No. contigs	104	95	79	129	100	100	108	82	111	97	95
GC contents (%)	38.84	38.82	38.84	38.83	38.82	38.82	38.84	38.84	38.84	38.82	38.82
Plasmids	0	0	0	0	0	0	0	0	0	0	0
Contig L50	11	15	8	10	15	16	12	8	9	15	16
Genome length (bp)	3,908,792	3,908,950	3,922,591	4,012,541	3,908,205	3,908,531	3,908,497	3,922,228	3,950,256	3,908,316	3,909,130
Contig N50	146657	97,227	166,466	137,936	97,227	93,542	130,594	162,218	126,319	97,227	95,517
chromosomes	0	0	0	0	0	0	0	0	0	0	0
RAST tool kit	470.11591	470.11589	470.1159	470.11592	470.11596	470.11595	470.11601	470.11598	470.11594	470.11593	470.11588
CDS	3809	3764	3804	3936	3768	3771	3809	3807	3859	3771	3765
rtRNA	58	58	59	59	60	61	57	62	56	61	61
rRNA	3	3	3	3	3	3	3	3	3	3	3
partial CDS	0	0	0	0	0	0	0	0	0	0	0
miscellaneous RNA	0	0	0	0	0	0	0	0	0	0	0
repeat regions	0	0	0	0	0	0	0	0	0	0	0
Pathogen. watch											
Genome length (bp)	3920736	3,922,856	3,931,803	4,022,278	3,922,411	3,921,255	3,921,639	3,931,813	3,961,239	3,919,669	3922482
No. contigs	90	92	61	107	91	90	93	63	92	85	91
Smallest contig	500	505	505	501	505	505	500	505	505	505	505
Largest contig	295270	219,760	432,764	364,686	220,110	220,042	230,466	432,764	354,726	219,521	154,013

Average contig length	43563	42,639	64,455	37,591	43,103	43,569	42,168	62,409	43,056	46,113	43,104
Contig N50	130744	94,042	166,280	130,743	94,042	94,042	125,444	166,364	118,767	97,377	94,042
NonATCG	600	500	700	800	900	800	800	900	900	700	600
GC contents (%)	38.80%	38.8	38.8	38.8	38.8	38.8	38.8	38.8	38.8	38.8	38.8
Organism prediction	470	470	470	470	470	470	470	470	470	470	470
Organism name	*Acinetobacter baumannii*	*Acinetobacter baumannii*	*Acinetobacter baumannii*	*Acinetobacter baumannii*	*Acinetobacter baumannii*	*Acinetobacter baumannii*	*Acinetobacter baumannii*	*Acinetobacter baumannii*	*Acinetobacter baumannii*	*Acinetobacter baumannii*	*Acinetobacter baumannii*
RefSeq reference	GCF_0020828885.1	GCF_001806385.1	GCF_001806365.1	GCF_001806425.1	GCF_001806385.1	GCF_001806385.1	GCF_002082885.1	GCF_001806365.1	GCF_001543995.1	GCF_001806385.1	GCF_001806385.1
Mash distance	0.0009591	0.0005542	0.0005542	0.0009591	0.0005542	0.0005542	0.0009591	0.0005542	0.0020494	0.0005542	0.0005542
Matching hashes	388/400	393/400	393/400	388/400	393/400	393/400	388/400	393/400	375/400	393/400	393/400
